# Multivariate analysis of energy and solar performance across Dubai: insights from MANOVA and cluster analysis

**DOI:** 10.1038/s41598-025-09730-4

**Published:** 2025-07-08

**Authors:** Mayyas Alsalman, Marwa Alraeesi, Ayman Alzaatreh

**Affiliations:** 1 Department of Industrial Engineering, American University of Sharjah, P.O Box 26666, Sharjah, UAE; 2 Deprartment of Mathematics and Statistics, American University of Sharjah, P.O Box 26666, Sharjah, UAE

**Keywords:** Electricity, Solar generation, MANOVA, Cluster analysis, Environmental social sciences, Climate-change impacts

## Abstract

Solar energy adoption became a key component in achieving the UAE’s sustainability strategy, featuring the abundance of solar irradiance in the region that tends to reduce the dependence on carbon-based resources through solar energy integration. However, despite the UAE’s solar energy adoption efforts, there is a clear gap due to the limited statistical analysis to evaluate the performance of such solar implementation across Dubai’s diverse community areas for the different building types. This study aims to investigate the performance of energy consumption and solar generation under the Shams Dubai program, specifically across the residential, commercial, and industrial sectors within various communities in Dubai. The study analyzed 93 community areas using hierarchal clustering Analysis and Multivariate Analysis of Variance to group and compare energy patterns. The clustering analysis identified three clustered groups that differ in building types and area, influencing energy consumption and solar energy generated, causing energy and solar pattern disparities. The MANOVA results confirmed a statistically significant difference with a 95% confidence interval across the three clusters. Accordingly, this study offers actionable insights for utility companies and policymakers to prioritize large-scale solar projects in high-demand commercial areas while focusing on underperforming residential areas by conducting awareness and incentive campaigns to enhance solar adoption. The study’s results enable more informed resource allocation, support progress toward Dubai’s solar adoption targets, and aid in developing tailored data-driven decisions for energy efficiency improvements. By translating statistical insights into practical strategies, this research allows decision-makers to reduce carbon emissions, optimize solar energy, and achieve sustainability objectives based on Dubai’s rapid urbanization context.

## Introduction

The UAE has witnessed continuous economic development driven by population growth and the industrial revolution in the past few years, leading to significant energy demand and raising environmental concerns due to the escalating carbon emissions^1^. The UAE’s reliance on carbon-based energy generation promoted the government to transform towards clean energy resources to achieve the sustainability agenda. Solar energy, in particular, offers a promising solution due to its abundant potential, especially in regions like the UAE; with its high solar radiation levels, exceeding 2200 kWh/m² annually, the UAE recognizes the vast potential of solar power in addressing its challenges of high energy consumption rates^2^.

In response to the environmental challenges, the UAE has set the 2050 clean energy strategy that aims to generate 50% of the country’s electricity needs from clean energy resources, mainly solar, by 2050, which will reduce carbon emissions by 70% and achieve the net zero by 2050. Given that 70–80% of the UAE’s electricity consumption is attributed to buildings, the country has significantly emphasized deploying solar technologies in residential, commercial, and industrial buildings, where solar energy systems are primarily installed in the UAE’s building sector^3^.

Dubai is a regional leader in implementing solar energy through large-scale and grid-tied integration for urban settings. Thus, the building sector accounts for 39% of Dubai’s energy consumption due to the high demand caused by HVAC cooling requirements. A significant example of supporting such a transition is the Shams Dubai initiative, launched to encourage Dubai residents to install solar PV panels on their rooftops and interconnect with the grid using the net-metering scheme. By 2023, the installed projects under the Shams Dubai initiative have reached an installed capacity of 601.8 MW^4^. In addition, Dubai aligns with the UAE’s sustainability development by integrating solar energy, which supports the UAE’s net-zero strategy, ensures an energy mix, and contributes to national carbon reduction.

However, despite the notable progress in solar energy adoption across the UAE and Dubai, the focus has been on large-scale power plants and pilot projects that overlook small-scale and building-integrated applications. A more comprehensive statistical analysis needs to be conducted that evaluates the comparative performance of solar systems across different sectors, where the focus on energy consumption and solar generation of these connected projects needs to receive more attention.

Moreover, the increasing installation of solar energy systems is well-documented. In contrast, limited research has been conducted to compare the performance of such systems across diverse categories of buildings, including residential, commercial, and industrial, within Dubai’s various community areas. Therefore, it is important to understand how solar enegry performs in diverse communities to optimize the emirate effective solar adaption. In addition, the varying energy consumption patterns based on the building types across different community areas can influence solar generation performance, and effectiveness research areas should be considered for improvements that aid valuable insights into further solar project investment plans.

This study fills the research gap by examining the performance of solar energy systems under the Shams Dubai program across different sectors and communities in Dubai. The study will use energy consumption and solar generation data to group communities based on similar energy consumption and solar performance patterns. The resulting clusters are then analyzed using MANOVA (Multivariate Analysis of Variance) to assess the statistical significance of differences in solar performance across these categories. The research findings can help utility decision-makers in understanding the solar projects performance and energy patterns and maximizing their energy generations and savings.

## Literature review

### Solar energy resource

The industrial revolution drastically increasing energy demand has impacted the environment by increasing carbon emissions to 75% from the energy sector alone. As such, the increase in energy demand has driven the global nations to shift towards sustainable and renewable energy resources, which play a vital role in reducing the effect of climate change by generating clean energy^5^. Renewable energy resources are clean energy replenished from nature that can be driven directly or indirectly from the sun^6^. However, six sources of clean energy, which include wind, solar, marine hydro, geothermal, and bioenergy, can be used to reduce emissions and diversify the energy mix. Solar energy, particularly solar photovoltaics, is a rapidly growing clean energy resource globally, accounting for 55% of the total investment in clean energy. Solar energy technology is the most versatile technology that plays a vast role in the clean energy transition in the world due to its high installed capacity in the building sector. Solar photovoltaics is also increasing in the GCC region due to the abundance of solar radiation^7^.

Moreover, countries have set strategies and policies to support grid-tied solar energy generation. Feed-in-tariff and net-metering have been successfully implemented worldwide in countries including Japan, Germany, UK, Italy, Spain, and USA. However, the GCC countries pledged to reduce their carbon emission by shifting towards solar energy generation to reduce their dependence on carbon-based resources^8^. The GCC countries implemented feed-in-tariffs and net-metering solar energy generation policy mechanisms to achieve their sustainability targets. The GCC countries are making crucial efforts to transition away from carbon-based resources. However, their 2030 energy targets included 50% of Saudi energy from clean energy, the UAE 30% and Oman will have 20% of their energy from solar resources^9^.

The UAE established itself as a leader in grid-tied solar energy in the GCC region through ambitious policy frameworks. Thus, Dubai Emirate stands out for it is well-addressed energy targets based on Dubai’s clean energy strategy for 2050 25% of energy would be from clean energy sources by 2030, which would scale up to 75% by 2050, with solar as a significant focus. Dubai’s efforts align with the UAE federal energy strategy 2050, targeting 44% of clean energy by 2050^10^. As such, the UAE’s goals are more explicit than those of other GCC regions; Bahrain, Qatar, and Kuwait have begun their policy deployment. Oman and Saudi have set clear targets, whereas their grid-tied solar energy policy implementation lags Dubai’s execution and maturity^11^. The next section focuses on the UAE’s solar energy deployment efforts.

### Adaption of solar energy in the UAE

**Fig. 1:**
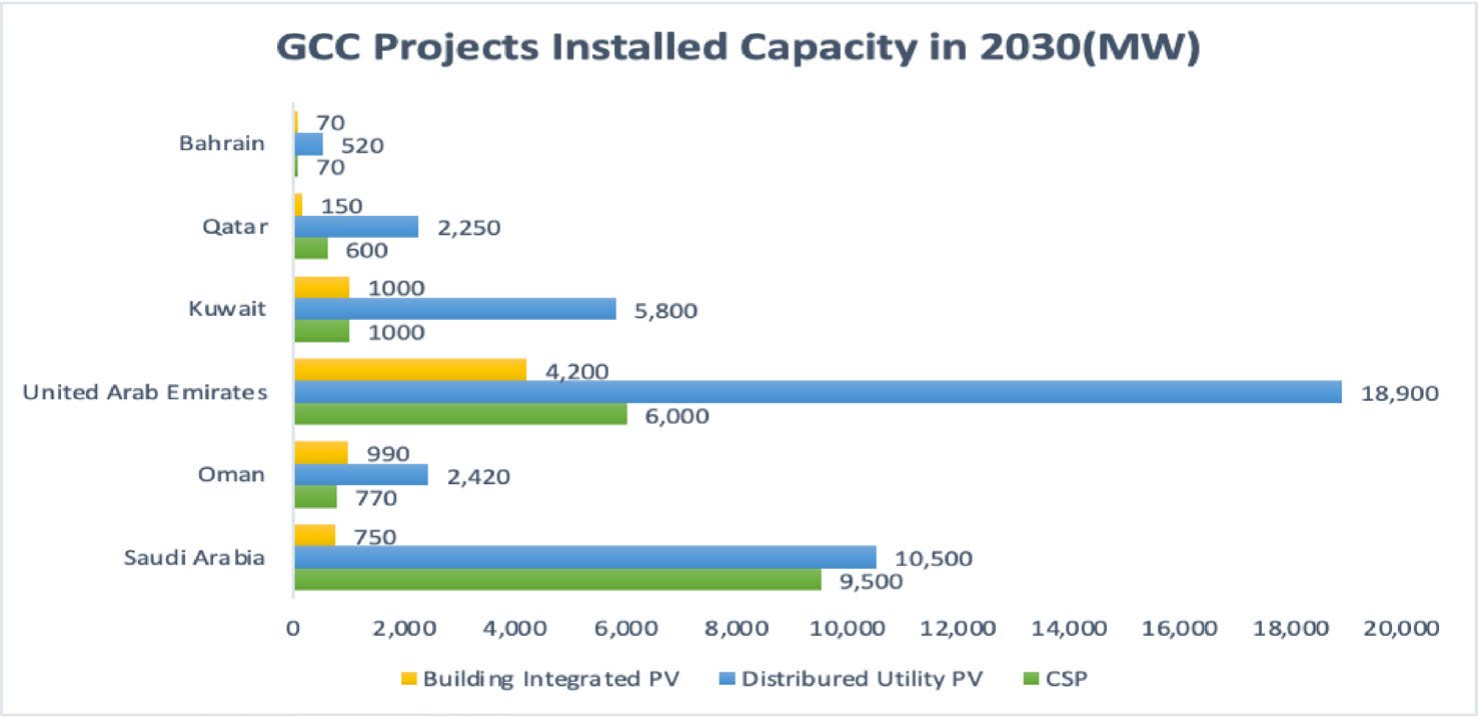
Projected Solar Installed Capacity in 2030 in the GCC Region, Adapted from^9^.

The UAE is a fast-developing country that has experienced impressive economic growth over the past few years, which increased energy consumption. The increasing consumption increased the UAE’s carbon emissions, making it one of the top 10 countries. Therefore, to address such an issue, the UAE listed climate change as one of its top priorities and initiated a clean energy strategy in 2050. Through this strategy, the UAE is committed to generating around 50% of its electricity demand from solar energy by 2050, reducing carbon emissions to 70%^12^. Also, the UAE is committed to reaching net zero by 2050, which supports shifting towards net zero buildings to reduce the amount of energy consumed, as the building sector alone accounts for 80% of total energy consumption^13^. In addition, the UAE has installed many utilities scale solar projects, making it the leading country in solar adaptation in the GCC region, as shown in Fig. 1.

The UAE is investing significantly in renewable energy projects to achieve its sustainability targets of producing 50% of the UAE’s energy needs from clean energy resources. The largest utility-scale project is Mohammed Bin Rashid Solar Park, which has an estimated capacity of 5 Gigawatt by 2030. Another utility-scale project is Shams 1 concentrated solar power initiated by Masdar Abu Dhabi with an installed capacity of 100 MW. Thus, the rooftop grid-tied solar installations are leading under the net metering scheme in the two emirates of Abu Dhabi and Dubai. Dubai launched its Shams Dubai Solar Generation initiative in 2015 for the different building types, whereas Abu Dhabi started rooftop installation in 2017 for governmental buildings^14^.

In addition, similar to international solar energy leaders, Dubai has established solar deployment targets through the grid connection policies focusing on large-scale and distributed solar, which led to making Dubai among the first globally to achieve a solar tariff rate under the net-metering below 3 US cents/kWh undercut the leading global solar markets such as Australia and Chile. However, Dubai’s regulatory framework features pioneering solar policy mechanisms due to the robust IPP model, transparent auctions for large-scale projects such as MBR solar park, and net-metering for distributed grid-tied solar through Shams Dubai, simulating commercial, industrial, and residential sectors^15^. Thus, Dubai’s solar policy deployment is facilitated by the Dubai Electricity and Water Authority to ensure efficient grid ties within the utility grid. Shams Dubai initiative will be further discussed in the next section.

### Shams Dubai solar energy generation

The building sector is the most promising application area for rooftop-installed solar photovoltaics, whereas it is estimated that around 40% of the world’s installed capacity is represented by rooftop installations. However, the advantage of rooftop installation is the easy implementation and the low levelized cost of electricity^16^. The rooftop installation in the UAE is tied to the utility grid using the net metering scheme, which contributes to achieving an energy mix, supporting the UAE Energy Strategy 2050 and aligning with the Sustainable Development Goal 7, emphasizing the importance of having reliable, sustainable energy resources for everyone. Moreover, the net metering supports the UAE’s distributed solar energy deployment by allowing consumers to reduce their electricity bills based on the project electricity tariff rate^17^.

Furthermore, Dubai Electricity and Water Authority (DEWA) launched the Shams Dubai Solar Energy Generation initiative in 2015, which allows customers to install solar photovoltaic modules on their rooftops to generate electricity that they can use directly. In contrast, the excess will feed back into the utility grid. Shams Dubai initiative supported various building types such as residential, commercial, and industrial. As of 2023, the amount of installed capacity has reached 601.8 MW. As such, grid-tied solar energy generation under Shams Dubai will provide consumers with a long-term reduced electricity bill, lowering energy consumption, and carbon emission, which aligns with the UAE’s strategy of achieving net zero by 2050^18^ .

## Methodology

This section outlines the approach of this study by detailing the data collection and statistical data analysis that will be conducted to explore the performance of solar generation in reducing electricity consumption across diverse building sectors: residential, commercial, and industrial within Dubai communities.

### Data collection

This study utilizes dataset of solar generation (kWh) and energy consumption (kWh) from 93 community areas across Dubai emirate, as shown in Table 1. The data was collected over multiple years since the solar integration initiative in Dubai, allowing for sectoral mapping of energy consumption and solar installation patterns across multiple urban zones and sectors. To ensure data reliability and validity, data pre-processing was undertaken, which included identifying missing data and removing duplicates.

**Table 1:** Dubai communities with solar Installations.

Sectors	Community Number	Community Areas
1	15	Nakhlat Deira, Al Muraqqabat, Port Saeed, Al Mamzar, Al Baraha, Al Buteen, Al Muteena, Al Ras, Al Rigga, Al Waheda, Hor Al Anz, Naif, Riggat Al Buteen, Tecom, Ayal Nasir
2	15	Muhaisnah, Mirdif, Al Mizhar, Oud Al Muteena, Al Eyas, Al Ttay, Al Garhoud, Al Ghusais. Al Khawaneej, Al Nahda, Al Rashidiya, Al Twar, Dubai Airport, Nad Shamma, Umm Ramool
3	31	Umm Hurair, Al Mankhool, Al Karama, Jumeirah, Al Bada’a, Al Satwa, Al Wasl, Al Merkad, Umm Suqeim, Al Qouz, Al Sufouh, Al Barsha, Nakhlat Jumeira, Al Thanayah, Al Hudaiba, Al Jadaf, Al Kifaf, Al Manara, Al Mina, Al Raffa, Al Saffa, Al Souq Al Kabeer, Al Thanayah, Bur Dubai, Emirates Hills, Jumeira Bay, Out Metha, Trade Center, Za’abeel, Umm Al Sheif
4	4	Al Khairan, Al Warqa, Ras Al Khor, Nadd Al Hammar
5	7	Mena Jabal Ali, Jabal Ali, Dubai Investment Park, Hassayan, Madinat Al Mataar, Jabal Ali Freezone, Saih Shauib
6	7	Nad Al Shiba, Warsan, Wadi Al Safa, Al Hebiah, Arabian Ranches, Me’aisem, Nadd Hessa.
7	3	Lehbab, Al Awir, Madinat Hind.
8	11	Al Rowaiyah, Margham, Hatta, Umm Al Mo’meneen, Umm Eselay, Al Yufrah, Al Layan, Al Lesaily, Al Marmoom, Saih Al Dahal, Umm Nahad

### Statistical analysis

Several statistical techniques will be used to assess solar integration using SAS on Demand for Academics Studio, whereas descriptive statistics will be used to analyze the amount of energy consumption and solar generation across the different building sectors, providing impactful insights. As such, hierarchal cluster analysis is selected to group the community areas in Dubai based on similarities in energy performance. The choice of clustering over other statistical analyses, such as principal component analysis (PCA), is driven by the research aim to identify communities based on similarities that could be viewed in the dendrogram to indicate the number of clusters. Thus, the one-way MANOVA (Multivariate Analysis of Variance) is selected over univariate ANOVA as it will assess the electricity utility in detecting the source of difference between the predefined cluster groups across Dubai building sectors. Also, one-way MANOVA tends to reduce the type 1 error, yielding a holistic insight from the multivariate energy patterns. The data analysis in this study will be used to analyze the solar energy generation and electricity consumption of 93 community areas throughout Dubai. Therefore, the data analysis will be divided into three stages: descriptive statistics, cluster analysis, and multivariate analysis of variance, which will be discussed in detail in the data analysis section.

### Data description analysis

Data description analysis is a foundational step in identifying key insights for further statistical evaluation and decision-making. To better understand the data distribution, a comprehensive SAS descriptive analysis is conducted primary on the amount of energy consumption and solar generation data, which is analyzed using box plots and correlation analysis as the following:

#### Box plot

**Fig. 2:**
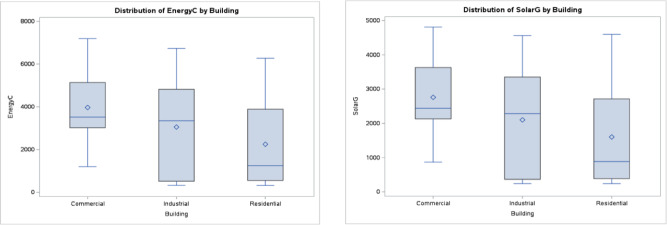
Box plot of Energy consumption across the three building types.

Box plots help visualize the spread and central tendencies of energy consumption and solar generation within each project building type (commercial, residential, or industrial), highlighting potential outliers and variations. By using the SAS analytical tool, the Fig. 2 is obtained:

The box for each sector shows that lower variability for commercial and residential sectors, indicating more consistent energy usage within these sectors. Thus, industrial sector has a wider box, suggesting greater variability in energy consumption. No outliers were evident in the box plot for any project type, meaning that all data points fall within the expected range for each sector.

#### Correlation analysis

**Fig. 3:**
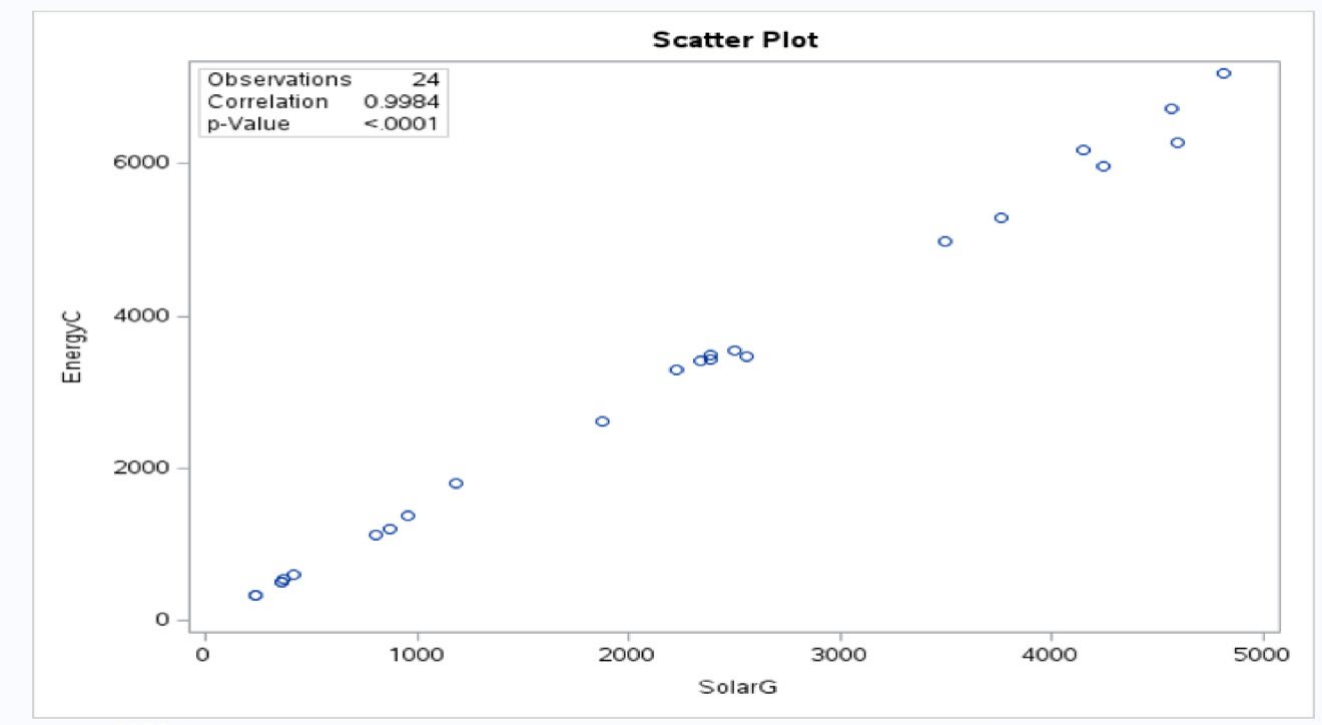
Scatter plot of energy consumption versus solar generation.

The following scatter plots were obtained to primarily explore the relationship between energy consumption and solar generation values and help visualize the correction between them as shown in Fig. 3.

However, the Pearson correlation analysis reveals a strong positive correlation, given = 0.99, indicating that solar energy generation also tends to increase as energy consumption increases. This strong positive relationship suggests that buildings with higher energy consumption are also equipped with larger solar generation systems with higher capacities or solar performance. However, it is important to understand that correlation does not necessarily imply causation. As such, higher energy consumption in specific building types does not necessarily cause an increase in solar energy generation. This highlights the need to consider the other underlying factors that could explain this relationship.

### Cluster analysis

Cluster analysis is a widely used statistical technique that groups data points into meaningful clusters based on their similarities, allowing researchers to identify patterns and relationships within complex datasets in different applications such as: earthquake analysis, climate studies, medicine, computer simulations, and energy resource management^19^. Different clustering analysis methods can be applied depending on the nature of data and the research objectives.

Hierarchical clustering is one of the key clustering approaches where a hierarchy of clusters is built by either merging or splitting them based on their similarities. It can be categorized in two types: agglomerative, where individual data points start as separate clusters and are gradually merged, or divisive, where all data points begin in a single cluster and are progressively divided into smaller ones^20^. In agglomerative hierarchical clustering, each observation begins as its own cluster, and the process involves repeatedly identifying the two most similar clusters and merging them until all clusters are unified.

Another key clustering approach is the K-means clustering, a technique that partitions data points into a specific number (K) of clusters by measuring their distances to the centroid of each cluster. This algorithm works to minimize the total sum of squared distances between data points and the centroids of their respective clusters^21^. The process of grouping data has garnered attention from both researchers and decision-makers, particularly in the energy sector, including government and public organizations. For example, K-means clustering is commonly used to segment communities based on energy usage patterns, while hierarchical clustering may be employed to explore hierarchical relationships within solar performance data. This study employs hierarchical cluster analysis though cluster observations and K-means functions in SAS On Demand software.

#### Cluster observation

Initially, Cluster Observation function employed the Complete Linkage method to determine the optimal number of clusters and categorize the sectors and building types. The criteria of a number of cluster plots obtained reveal the CCC, the Pseudo-F, and the Pseudo T-Squared criterion method. By analyzing the local max of the CCC, the Pseudo-F, and the local mins of the Pseudo T-Squared criterion method, the results indicate an optimal number of 3 clusters representing the various communities or groups in Dubai.

**Fig. 4:**
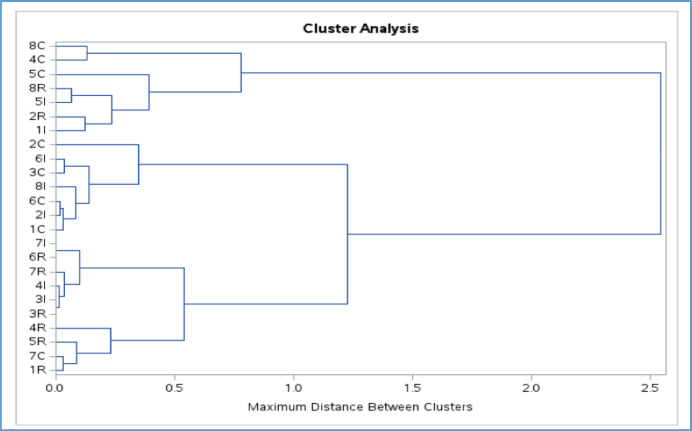
Dendrogram plot using complete linkage method.

Similarly, the cluster analysis plot (Dendrogram) obtained supports retaining three clusters. The clear separation suggests that the data points within each cluster are similar to each other than to those in other clusters, as outlined in a vertical line were crossed, as shown in Fig. 4. The 3-cluster solution was also verified by various statistical measures such as Silhouette and cubic clustering criterion (CCC).

### K-means clustering

**Fig. 5:**
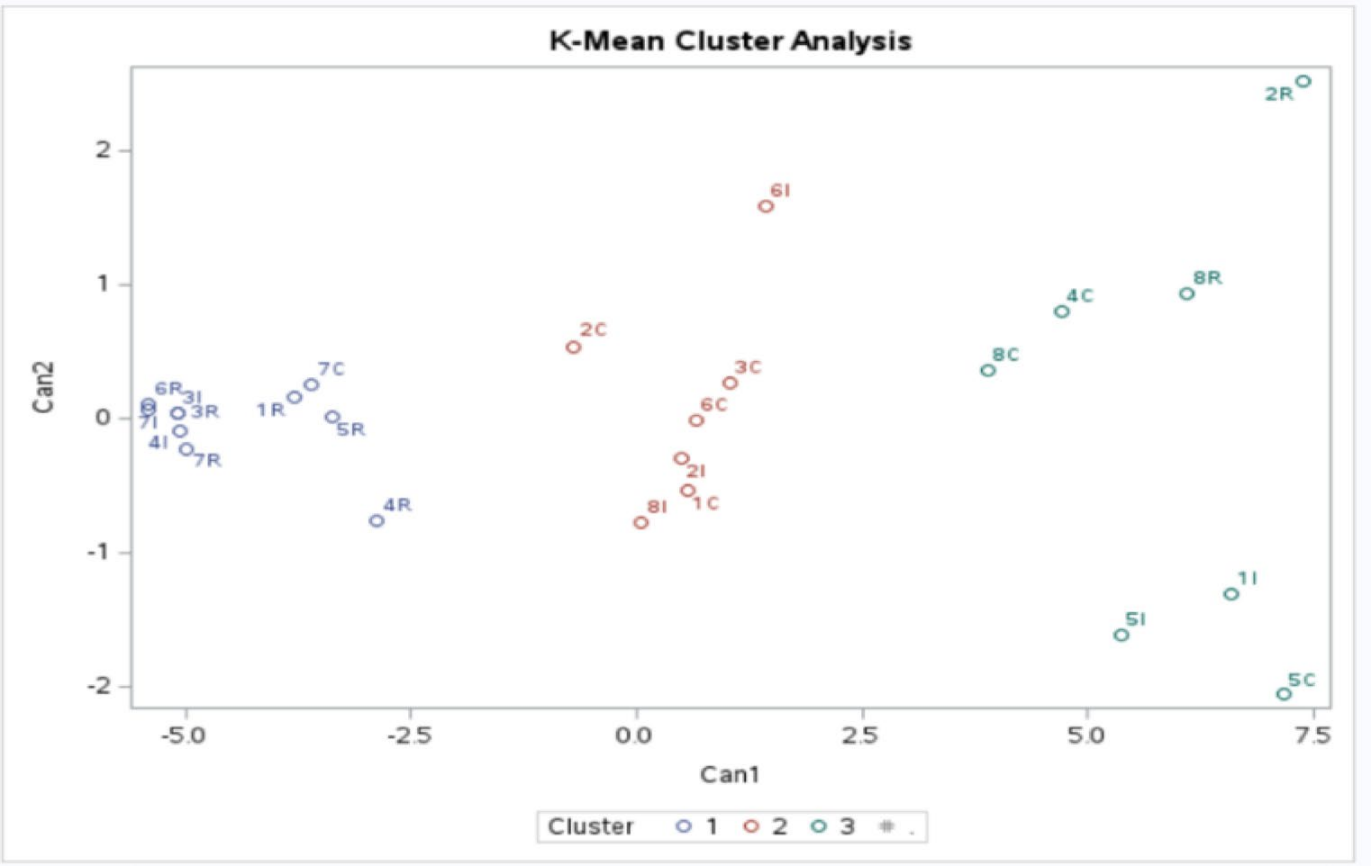
K-means cluster Analysis plot.

Three-cluster solution was also verified by the k-means clustering method. A k-mean cluster analysis plot was obtained to visualize the different clusters better. The plot displays the results of clustering Dubai’s energy consumption and solar generation data into three groups, with each cluster represented by a distinct color, as shown in Fig. 5.

#### Clustering results interpretation

The core findings of the clustering analysis are summarized in Table 2, which tends to categorize the community areas in three clusters based on their energy profile.

**Table 2:** Clustering interpretation summary.

Cluster	Cluster observation	Findings	Characteristics
Cluster 1	7I, 3I, 4I, 6R, 3R, 1R, 5R, 4R, 6R,7R,7 C	Mostly residential areas	Moderate to low energy consumption, Low Solar generation
Cluster 2	6I, 8I, 2I, 2 C, 1 C, 6 C, 3 C	Mostly commercial projects, areas close to the downtown of Dubai	Moderate energy consumption, Moderate Solar generation
Cluster 3	8 C, 4 C, 5 C, 5I, 1I, 8R, 2R	Mostly commercial, Dubai boarders	High energy consumption, High Solar generation

The projects in Cluster 1 are characterized by moderate to low energy consumption and low solar energy generation compared to Cluster 2, which shows Moderate Energy consumption and moderate Solar generation. Cluster 3 includes the projects with the highest energy consumption and solar generation among all the projects in Dubai. With these clear groupings, policymakers and energy providers can make more informed decisions as the obtained output makes it easier to understand cluster-specific behaviors to address informative solutions for energy consumption and solar performance. In this context, the cluster findings can be interpreted as the following:

- **Cluster 1**: Moderate to low energy consumption and Low Solar generation.

The cluster represents possibly industrial sectors and primarily residential buildings that might lack solar installations. The utilities can find opportunities for industrial sectors to adopt larger-scale solar installations, given that most industrial facilities include significant rooftop or unused land space. However, the lack of solar installations suggests that these industries might be unaware of the benefits or not yet incentivized to utilize these large rooftops. Similarly, there might be a lack of public awareness or willingness to invest primarily in solar systems for residential buildings. Smaller rooftops and limited knowledge about the feasibility or affordability of a solar energy system may limit adoption. This cluster might benefit from awareness campaigns focusing on the key benefits of solar power, such as long-term savings, environmental impact reduction, increased property value, and reduced footprint. Hence, residents could be motivated to explore solar installation options.

- **Cluster 2**: Moderate Energy consumption and Moderate Solar generation.

This cluster includes areas near downtown Dubai, which are known for their high activity level. These areas demonstrate moderate energy consumption and solar power levels, mainly due to the limited roof space in this dense region, which is mostly built up with towers, leaving limited space for large-scale projects and, therefore, larger generation capacity. On the other hand, those areas have been the focus of the city’s development initiatives, aiming to showcase the city’s commitment and potential targets of clean energy by strategically deploying solar in the available green spaces, incorporating solar in further applications such as in streetlight, and offering free solar installation to the residential buildings in this region to overcome the challenges posed by urban density. Incorporating energy-saving and further renewable energy solutions is suggested in this area to utilize the full benefit of solar energy and to consider both the constraints of limited rooftop space and the city’s ongoing efforts to showcase solar energy potential.

- **Cluster 3**: High energy consumption and high solar generation.

This cluster likely represents commercial projects, primarily located on the borders of Dubai. The high solar radiation in this area suggests that these projects have large solar capacity, likely due to their large rooftop areas. The location near Dubai’s borders provides more solar panel space than the more densely built-up areas closer to the city center. The high energy consumption observed in these projects indicates a significant energy demand, likely due to the scale of operations in this region. As a result, these projects are excellent candidates for possible solar incentives due to their high solar generation capacity. Additionally, implementing energy management systems should be prioritized for the projects in cluster 3 to optimize energy efficiency further.

Moreover, socio-economic, geographic, and policy incentives drive solar adoption across Dubai community clusters. The residential community in cluster 1 has a low-rise building with limited roof space, resulting in lower solar generation. In contrast, such areas have diverse communities with different awareness and willingness to invest in solar energy. Also, residential buildings have lower energy consumption than commercial and industrial buildings, which impacts the low-income community’s investment in solar energy despite its long-term electricity-saving potential. Thus, by looking into the geographic factors cluster 2, which are communities across the Downtown and Dubai borders with moderate solar generation despite their limited roof space, such areas could benefit from the available infrastructure for solar implementation and effective energy-efficient technologies that improve the overall energy efficiency. Cluster 3 has significant rooftop areas that could benefit from a larger-scale solar installation.

In addition, the geographical location of the different clusters also plays a major role, as communities across Dubai’s borders have higher solar radiation due to less shading, which enhances solar effectiveness. For the Shams Dubai net-metering policy, the different clusters could benefit from a lower electricity cost that is the same for all clusters.

### Multivariate analysis of variance

Multivariate Analysis of Variance (MANOVA) is defined as the statistical technique used to test the differences in dependent variables across multiple groups. The MANOVA is a generalization of the univariate ANOVA with more than one dependent variable^22^. MANOVA is more appropriate than multiple ANOVA as it will avoid type 1 inflation error that can be controlled by using Bonferroni adjustment and post-hoc comparisons, whereas MANOVA also considers the correlation between different response variables^23^.

As such, the purpose of MANOVA is to determine whether the independent variables have an effect on the dependent variable within the model being tested. In addition, MANOVA can also detect patterns between multiple variables when they are analyzed together. There are two types of MANOVA, the one-way MANOVA is used to test the effect of a single independent variable on multiple dependent variables to examine if they have a significance difference in one or more characteristics. In comparison, the two-way MANOVA is used to test the effect of two independent variables on multiple dependent variables by including any interaction effect between the variables, which makes it more complex^24^ .

Moreover, there are several applications of MANOVA and ANOVA in literature, whereas ANOVA was used in^25^ to detect the influence of energy consumption in smart homes in Germany. In^26^, this study used ANOVA and MANOVA statistical analysis to explore the effect of different variables on solar irradiance in Brazil. Another study^27^ used MANOVA to investigate the relationship between the economic awareness of the Polish household and the effect of their behavior on energy consumption by considering several factors.

In this study, one-way MANOVA is used to investigate the significant differences between the community sectors and building types, which are grouped into three clusters to investigate their effect on solar generation and energy consumption. The independent variables are the clusters (cluster 1, cluster 2, and cluster 3), whereas the dependent variables are the electricity consumption (kWh) and solar energy generation (kWh). The validity of MANOVA assumes normality that is tested and confirmed based on the* p*-values for Mardia Skewness and Marida Kurtosis; 0.0910 and 0.4898 are greater than 0.05, and the residuals on the Q-Q plot supports the normally assumption. In contrast, the second validity test is the variance-covariance homogeneity that is confirmed based on the* p*-value of the Box-test of 0.2169, which is greater than 0.05.

#### Analysis of the impact of cluster on solar generation and energy consumption

To investigate the significance of predefined clusters on solar performance and energy consumption the null hypothesis was mainly the equality of mean among the three clusters. While the alternate hypothesis stated at least one of the three clusters is different. According, to Wilk’s Lambda* p*-value 0.001, which is less than 0.05 this indicates that there is a difference between solar generation and energy consumption among the clusters.

#### Testing the differences between cluster groups

After finding the overall significance of the clusters the post-hoc pairwise test is conducted to investigate the difference between the three clusters in Dubai. As a result, the pairwise comparison of the average differences between the cluster groups showed that there is a difference in the amount of solar generation and electricity consumption between the cluster groups. Since, there is a difference between all clusters in terms of the amount of solar generation and energy consumption, it is important to construct a 95% Bonferroni simultaneous confidence interval for the average differences. As such, the Bonferroni simultaneous confidence interval is conducted to estimate the average difference between the cluster groups, whereas it is constructed for cluster 1 versus cluster 2, cluster 1 versus cluster 3 and cluster 2 versus cluster 3 for both energy consumption and solar generation as shown in Table 3.

**Table 3:** Bonferroni simultaneous test for mean Difference.

	Comparison	Difference Between Means	Simultaneous 95% Confidence Limits	
			Lower Limit	Upper Limit
Electricity Consumption	Cluster 1 - Cluster 2	0.40961	0.24074	0.57847
	Cluster 1 - Cluster 3	0.24176	0.08607	0.39745
	Cluster 2 - Cluster 3	0.65136	0.49567	0.80705
Solar Generation	Cluster 1 - Cluster 2	0.34938	0.22904	0.46971
	Cluster 1 - Cluster 3	0.39414	0.28320	0.50508
	Cluster 2 - Cluster 3	0.74352	0.63258	0.85446

Table 3 showed that the solar generation and energy consumption for different clusters differed. As such, the MANOVA test and multiple comparisons for electricity consumption revealed that the confidence interval differed significantly in cluster 1 from the other two clusters, which had higher average values. The highest mean difference is between Clusters 2 and 3 (0.65136), whereas the lowest is between Cluster 1 and Cluster 3 (0.24176). Moreover, for solar generation, the highest mean difference is between cluster 2 and cluster 3 (0.74352), and the lowest is between clusters 1 and 2 (0.34938). In general, for both electricity consumption and solar generation, cluster 1, primarily residential areas, showed the lowest values compared to others.

Therefore, MANOVA analysis revealed the significance of the cluster group effect on energy consumption and energy generation across Dubai’s various community areas. For electricity consumption, cluster 3 is the highest, followed by clusters 2 and 1, whereas for solar generation, cluster 3 is the highest, followed by clusters 1 and 2. This indicates that the electricity utility should make more efforts to address the amount of energy consumed by Cluster 3 and Cluster 2, which are primarily commercial buildings. Also, more solar installations should be considered in Cluster 1, residential buildings, and Cluster 2, commercial buildings, as they have lower solar generation to achieve Shams Dubai’s solar target across Dubai community areas for the different building types. Moreover, the results reflect Dubai urban planning and zoning, whereas cluster 1 typically involve low rise buildings with less rooftop space available, cluster 2 downtown areas have limited spaces but available infrastructure that support the solar installations. While, cluster 3 has more available land and more rooftop availability such differences tend to boost the solar installation.

## Discussion

Throughout this study, the were investigated in 93 community areas around Dubai using Hierarchy cluster analysis to gain a deeper understanding of solar energy generation and electricity consumption and identify groups of sectors with similar consumption and generation patterns across different building types. In the same context, different studies have used cluster analysis to analyze renewable energy assets to optimize efficient resource in^28^ and the use deep learning and clustering to enhance the solar radiation forecasting accuracy^29^. In Dubai, where energy consumption and solar power adoption vary widely across different sectors and communities, cluster analysis is particularly valuable in identifying regions with similar characteristics, which can inform targeted decision makers of energy efficiency and solar energy deployment. According to^30^, clustering analysis were used to compare the utilities consumptions in Dubai as per category and community which can be similarly analyzed to study solar energy.

Therefore, the use of hierarchical cluster analysis in this study is well-suited for understanding the complex patterns of energy consumption and solar generation in Dubai. By grouping sectors with similar characteristics, this approach enables a more targeted analysis of energy behaviour across different building types and regions, which can inform strategies for improving energy efficiency and enhancing solar energy adoption. Additionally, it offers valuable insights for decision-makers to optimize resource allocation and implement policies tailored to specific areas, ultimately supporting the transition to more sustainable energy practices in Dubai.

The clustering analysis has provided a clear understanding of the varying energy consumption and solar generation patterns across different projects in Dubai. Cluster 1, characterized by moderate to low energy consumption and low solar generation, highlights opportunities for raising awareness and encouraging solar adoption in industrial and residential sectors. Cluster 2, with moderate energy consumption and solar generation, suggests that energy-saving solutions can be effectively implemented in more active urban areas to optimize solar energy utilization. Meanwhile, Cluster 3, which exhibits high energy consumption and high solar generation, presents commercial projects located on the borders of Dubai, where large rooftop spaces and high solar radiation offer significant potential for large-scale solar applications. These findings assist each cluster, ensuring more targeted and effective approaches to enhancing energy efficiency and solar performance across Dubai.

In addition, this study used one-way MANOVA to investigate the overall significant differences between the community sectors and building types that are grouped in three clusters to investigate their effect on the dependent variables: solar generation and energy consumption. The result of the MANOVA analysis reflected a significant difference between the three clusters, and it is important to understand such a difference as it can help the electricity utility focus its efforts on monitoring the amount of electricity consumption by increasing solar generation in certain areas. This result is consistent with a finding conducted by^30^, who compared the utility consumption in the emirate of Dubai for different community areas using MANOVA analysis.

Moreover, understanding the significant differences between the different clusters can help the electricity utilities monitor their energy patterns. As such, MANOVA multiple pairwise comparisons and Bonferroni simulations confidence interval revealed the effect of the cluster on the energy pattern across Dubai’s various community areas. These pairwise comparisons align with previous research comparing the different consumer building types, demand and renewable energy source^31^. The results of MANOVA imply that the electricity consumption is higher in cluster 3, followed by clusters 2 and 1, whereas for solar generation, cluster 3 is the highest, followed by 1 and 2. Therefore, the MANOVA result indicates that the electricity utility should address the increasing electricity consumption in clusters 3 and 2, primarily commercial buildings. In contrast, more solar installations are required in Cluster 1 residential buildings and Cluster 2 commercial buildings.

Therefore, identifying the energy consumption and solar generation patterns across Dubai’s communities in three clusters informs decision-makers in tailoring solar incentives for the different buildings. They should prioritize policy-driven incentives from the net-metering and feed-in-tariffs for the large-roof buildings. The underperforming clusters may be offered low-interest solar investment loans to enhance their solar implementation. Also, decision-makers should focus on targeted awareness campaigns since the residential cluster had limited awareness; such campaigns should demonstrate the economic and environmental benefits of the grid-tied solar energy adaption. Moreover, it offers consumers digital software to track their energy consumption and calculate their electricity savings due to solar generation, which drives the transition towards solar, reinforcing Dubai’s leadership in sustainable deployment.

Similar to any other study, this research encountered limitations that included the lack of previous studies focusing on comparing the effect of the building sectors on energy consumption and solar generation within the UAE region. This study depends on the energy results based on the available data that did not involve all the factors influencing the amount of energy consumption and solar generation, such as seasonal variations, energy slab rate, and solar generation technology. In addition, the statistical analysis did not explore the potential of future optimization within each cluster, which might include considering external factors such as economic and population growth within the different community areas across Dubai that impact future energy needs and solar generation capacity. However, understanding such factors might aid in generalizing the findings of this study across the UAE’s energy utilities by reducing the impact of energy consumption on the environment, which will cause the creation of comprehensive clean energy strategies.

Furthermore, including additional factors in future research, such as seasonal variations and solar energy generation policy, can aid in providing a better understanding of energy utilization and solar generation that might include suggesting additional statistical methods for energy analysis. In addition, expanding the dataset by considering a broader range of projects, including smaller residential projects and emerging commercial and governmental buildings, could help redefine the clustering approach and MANOVA significance. Also, selecting different dependent and independent variables for the MANOVA analysis might give different insights into the electricity utilities.

## Findings broader implication

This study focuses on Dubai’s grid-tied solar performance, whereas the findings could be generalized to urban contexts in regions perusing rapid grid-tied solar energy integration. The GCC countries, for instance, have launched their grid-tied solar programs, yet the UAE and specifically Dubai have set clear policy frameworks and solar invectives, causing it to lead. On a global scale, Germany, the United States, Japan, and India have implemented a net-metering scheme. In contrast, Dubai’s solar tariff competitiveness reaches a lower tariff rate below 3 US cents/kWh, making it favorable compared to leading international countries. However, researchers and decision-makers worldwide could adopt the clustering and MANOVA analysis to segment building types across community areas in similar clusters to understand their solar energy behavior and enhance the solar strategies based on data-driven insights in mixed urban clusters. Also, based on the people’s awareness and financial incentives policy, makers could enhance their emerging solar deployment. Therefore, the findings of this study offer a scalable statistical analysis approach for diverse regions to accelerate their solar adoption through generalized frameworks in emerging and developed countries to align with their sustainability targets and agenda.

## Conclusion

This research focused on addressing the high emissions due to the rapidly increasing energy consumption resulting from the UAE’s rapid economic growth and industrial revolution. Based on the UAE’s geographical location, the country has a high potential for integrating solar energy solutions due to the abundance solar irradiance in the region to lower the dependence on conventional resources and shift towards clean energy. In response, the emirate of Dubai launched the Shams Dubai program to encourage consumers to install photovoltaic modules on various buildings across Dubai’s various areas. However, despite the efforts of solar integration, there is a critical need to understand the performance of such projects across the emirate.

Throughout this study, several statistical methods using SAS software were used to analyze the performance of solar projects and energy consumption under the Shams Dubai program for different building types: residential, commercial, and industrial distributed in 93 community areas across eight sectors. Using the descriptive statistics, the box plots were found for each sector whereas the data is narrower for commercial and residential sectors, indicating more consistent energy usage within these sectors. Thus, industrial sector has a wider box, suggesting greater variability in energy consumption. In addition, the correlation analysis was found and indicated a strong positive correlation of 0.99, whereas correlation does not imply causation, as high consumption in buildings does not necessarily have a high solar generation.

As such, by using the hierarchy clustering analysis, 93 community areas were clustered into three groups based on their similar energy consumption and solar generation patterns. Cluster 1, primarily residential buildings, was found to have moderate to low energy consumption and low solar generation. Cluster 2, on the other hand, primarily involves industrial and commercial projects in areas close to downtown Dubai, with moderate energy consumption and solar generation. Whereas Cluster 3, primarily commercial on Dubai borders, has a higher energy consumption and solar generation. Based on the cluster analysis findings, policymakers and energy utilities can decide to monitor the energy patterns based on the cluster behavior to address the high energy consumption in the region.

One-way MANOVA was used to investigate the difference between the clusters, and the result reflected that there was a significant difference between the three clusters in terms of their energy patterns, which aligned with the clustering analysis findings. MANOVA indicated that the electricity utility should address the increasing electricity consumption in Clusters 3 and 2, primarily commercial buildings. At the same time, more solar installations are required in Cluster 1, which is residential buildings, and Cluster 2 commercial buildings. Therefore, the outcome of this study could be used by energy utilities to address and monitor the energy patterns of different buildings based on their similarities. However, for future studies, additional factors should be considered to aid in generalizing the findings across UAE energy utilities.

## Data Availability

The datasets used during the current study are available from the corresponding author on reasonable request.
